# The Diagnostic Accuracy of Pure-Tone Audiometry Screening Protocols for Vestibular Schwannoma in Patients with Asymmetrical Hearing Loss—A Systematic Review and Meta-Analysis

**DOI:** 10.3390/diagnostics12112776

**Published:** 2022-11-14

**Authors:** Liliana Gheorghe, Dragos Negru, Mihail Dan Cobzeanu, Octavian Dragos Palade, Eosefina Gina Botnariu, Bogdan Mihail Cobzeanu, Maria-Luiza Cobzeanu

**Affiliations:** 1 Surgical Department, Faculty of Medicine, University of Medicine and Pharmacy “Grigore T. Popa”, 700115 Iasi, Romania; 2 Emergency Clinical Hospital “Sfântul Spiridon” Iasi, 700111 Iasi, Romania; 3 Internal Medicine Department, Faculty of Medicine, University of Medicine and Pharmacy “Grigore T. Popa”, 700115 Iasi, Romania; 4 Clinical Rehabilitation Hospital, 700661 Iasi, Romania

**Keywords:** vestibular schwannoma, acoustic neuroma, pure-tone audiometry, magnetic resonance imaging

## Abstract

(1) Background: Magnetic resonance imaging (MRI) is the gold standard investigation for all patients who present with asymmetrical hearing loss (AHL) and a high index of suspicion for vestibular schwannoma (VS). However, pure-tone audiometry (PTA) is an investigation that can be used for the screening of these patients in order to reduce the costs. The aim of this systematic review and meta-analysis was to evaluate the diagnostic accuracy of different PTA protocols for VS in patients with ASHL, when compared with MRI; (2) Methods: Medline, Embase, and Cochrane databases were used to find relevant studies. All prospective and retrospective observational studies that evaluated the accuracy of PTA protocols for the screening of VS were assessed, according to the international guidelines; (3) Results: We analyzed seven studies (4369 patients) of poor-to-moderate quality. Their pooled sensitivity was good (0.73–0.93), but their specificity was low (0.31–0.60). All protocols were located in the right lower quadrant on the likelihood scattergram, and the post-test probabilities for positive and negative diagnosis of these protocols were extremely low; (4) Conclusions: PTA protocols cannot be used for a proper screening or diagnosis of vestibular schwannoma despite their good sensibility, and MRI remains the gold standard for this purpose.

## 1. Introduction

The Schwann cells of the vestibular (8th cranial) nerve give rise to the benign tumor known as vestibular schwannoma (VS)/acoustic neuroma (AN). Despite their benign character, these tumors have the ability to grow and can cause severe ontological symptoms, such as unilateral sensorineural hearing loss/asymmetrical hearing loss (AHL), vertigo, and tinnitus, due to impairment of the vestibulocochlear nerve function [1,2]. Gradual, high-tone hearing loss with higher asymmetry in the frequency range of 2–8 kHz is a typical characteristic of VS [3]. Headaches, visual changes, hypoesthesia, and palsies are just a few of the additional symptoms that could manifest as a result of a VS [4,5].

A VS typically grows between 0.99 and 1.11 mm every year, but certain characteristics of the tumor, including cystic and hemorrhagic appearances, as well as erythropoietin treatment, have been proven to indicate an accelerated growth [6]. 

Regarding the epidemiological profile of VS, it was demonstrated that the radiologically confirmed vestibular schwannoma rates increased in recent years in the United States of America (2006–2017: annual percentage change—1.7%; 95% confidence interval, CI: 0.5–3.0%) [7]. A retrospective longitudinal study that evaluated the incidence of acoustic neuroma in Iceland for a time frame of 30 years indicated an incidence rate of 1.24/100,000, as well as an ascending trend in the diagnosis of this condition [8]. At the same time, a recent systematic review that assessed the global incidence of sporadic vestibular schwannoma on four distinct populations from Denmark, the Netherlands, Taiwan, and the United States reported an incidence rate ranging from 3.0 to 5.2 per 100,000 person years, as well as an increased lifetime prevalence of sporadic vestibular schwannoma (>1 per 500 persons) [9]. Moreover, it appears that the age of the patient at the time of diagnosis of VS has been slowly increasing from 49 years in 1976 to 58 years [10]. 

Although a consistent association between long-term mobile phone use and the risk of developing VS has not been documented, there is heterogeneity within investigations, and higher risks have been noted in several studies for use of more than 10 years [11,12,13]. Exposure to high doses of radiation and mutations of tumor suppressor genes, such as neurofibromatosis 2 (NF2) gene, were linked to the development of sporadic or genetic variants of the disease [14,15]. 

Magnetic resonance imaging (MRI) is the gold standard investigation for all patients who present with asymmetrical hearing loss [16]. The use of MRI in the diagnosis of VS was the subject of a systematic review and cost-effectiveness analysis by Fortnum et al. [17]. Despite the fact that gadolinium-enhanced T1-weighted MRI is considered as the gold standard, there was little difference between it and non-contrast T2-weighted scans in terms of sensitivity and specificity. Additionally, non-contrast T2-weighted scans were thought to be more affordable for use in clinical settings.

However, screening methods have been devised to save and maximize resources because the number of MRI exams required for this group of patients is very high, and the number of schwannomas discovered is relatively low. An objective approach is represented by audiometric protocols based on quantifying the pure-tone audiometric (PTA) threshold by the difference in “decibel” and “frequency regions” between two ears [18]. Currently, there are multiple PTA protocols described in the literature, reported to have variable sensitivities and specificities for the diagnosis of VS depending on their definition of interaural asymmetry. 

The aim of this systematic review and meta-analysis was to evaluate the diagnostic accuracy of different PTA screening protocols for vestibular schwannoma in patients with asymmetrical hearing loss and to compare it with the gold standard represented by MRI. 

## 2. Materials and Methods

We performed a systematical search of published studies that evaluated PTA protocols for the screening of VS, in comparison with MRI examination in MEDLINE, EMBASE and Cochrane Library using synonyms of ‘magnetic resonance imaging‘, ‘asymmetrical hearing loss‘, ‘vestibular schwannoma‘, and Boolean operators AND/OR in accordance with Preferred Reporting Items for Systematic Reviews and Meta-Analyses (PRISMA) guidelines (Supplementary Material S1–S3: Search strategy). This systematic review and meta-analysis is registered in the Open Science Framework Registry (DOI: 10.17605/OSF.IO/FRGTC (accessed on 9 October 2022)).

The time frame settled for this research was from the beginning of the databases up to the first of September 2022, and we applied English language restriction as a filter. Additional research consisted of manual screening of references cited in the evaluated papers in order to ensure that all relevant studies were included. Duplicates were removed using EndNote software version 20.4 (Clarivate, Philadelphia, PA, USA). The full-text papers were independently reviewed by two investigators (M.D.C and D.N.) to establish their eligibility for the review. Any differences between the two were remedied by a third reviewer (M.L.C.) if a consensus could not be reached. 

The inclusion criteria were represented by observational studies, both prospective and retrospective, with a diagnostic study design that compared at least one PTA screening protocol to MRI findings in patients with AHL and comprised sufficient data for a 2 × 2 contingency table creation. We excluded opinion papers, animal studies, and case reports from the search. 

Two investigators (B.M.C and M.L.C) retrieved data from the eligible studies separately using a standard process. Data concerning the first author, publication year, study design, characteristics of the population examined, number of cases and controls, cut-offs used, and the information needed to create a 2 × 2 table were obtained. Two independent reviewers (B.M.C and M.L.C) assessed the methodological quality of the included studies using the QUADAS-2 technique (Quality Assessment of Diagnostic Accuracy Studies-2) [19]. Any disagreements [2] were resolved by discussion with a third reviewer (L.G.).

We summarized data from each study in 2 × 2 tables of true-positive, false-positive, true-negative and false-negative values, and we calculated sensitivity, specificity, and positive and negative likelihood ratios, as well as diagnostic odds ratio. For hierarchical modeling, a hierarchical summary receiver operating characteristic (HSROC) model will be utilized to generate equal summary estimates for sensitivity and specificity, taking into account variability both between and within studies (heterogeneity). In order to show variation and explore heterogeneity for sensitivity and specificity, we drew Forrest plots, likelihood ratios scattergrams, bivariate boxplots, and Fagan nomograms. I^2^ statistic was used to quantify the degree of heterogeneity. The statistical analyses were performed using STATA SE (version 14, 2015, StataCorp LLC, College Station, TX, USA). 

## 3. Results

**Figure 1:**
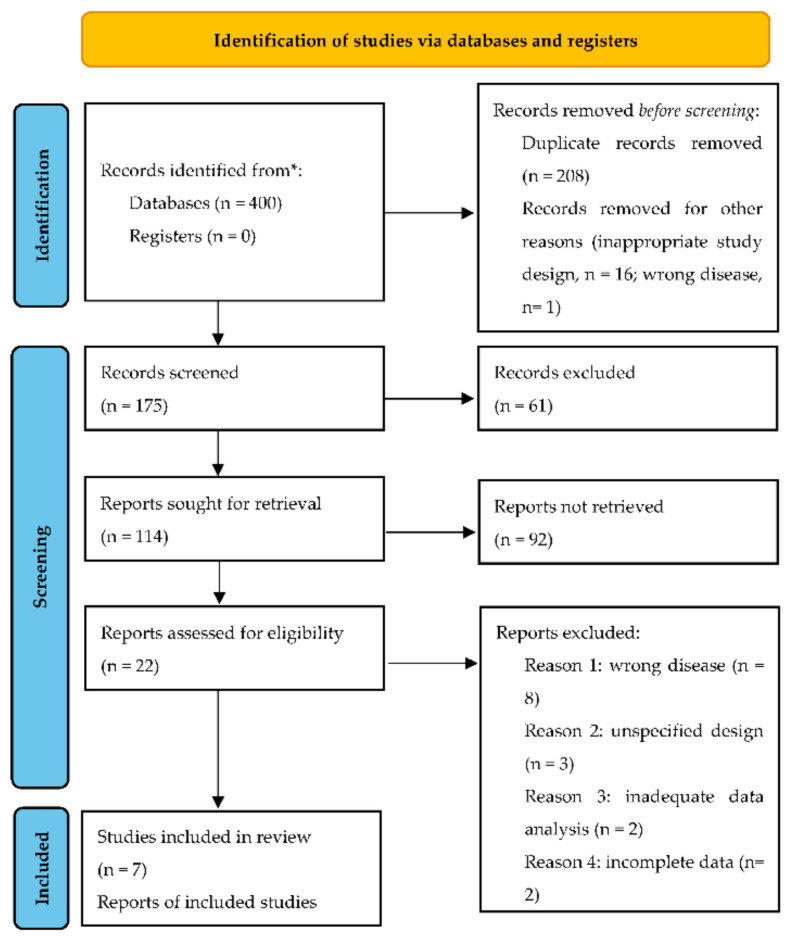
Flow diagram of search and selection of studies. * markup of data source.

Our search yielded 400 unique records, out of which only 7 were included in the meta-analysis after abstract and full text screening (Figure 1). We did not retrieve additional items after screening references and related articles. 

The characteristics of the included studies are presented in Table 1. A total of 4369 patients and 11 PTA protocols were included for further analysis. For the purpose of this meta-analysis, we evaluated the diagnostic accuracy of PTA protocols that were evaluated at least four times in the included studies mainly because the statistical analyses were not informative when using insufficient data. The included PTA protocols and their definition of asymmetrical hearing loss are presented as supplementary material (Table S1—Definitions of the included PTA protocols). 

Overall, the quality of included studies was low-to-moderate (Table 2). Two studies found a high risk of bias in one domain (patient selection) [24,25]. For the rest of the domains, the risk of bias was assessed as low and unclear. For the domains, patient selection, index test, and reference standard, respectively, none of the included studies scored highly on concerns regarding applicability. For the majority of the articles, there was little concern that applicability of the articles did not fit the review question. No studies were excluded from the analysis based on the quality. 

The pooled estimates and confidence intervals of sensitivity, specificity, positive and negative likelihood ratios, and diagnostic odds ratio, corresponding to the evaluated PTA protocols are presented in Table 3. 

The highest pooled sensitivity was achieved by the following protocols: Mangham 0.93 (95% CI: 0.76–0.98) [18,21,23,24,25], Amclass 0.93 (95% CI: 0.89–0.95) [18,22,23,24,25], and Nashville 0.91 (95% CI: 0.86–0.94) [18,20,22,24,25], while the highest pooled specificity and pooled positive likelihood ratio was achieved by the American Academy of Otolaryngology protocol (AAO) at 0.60 (95% CI: 0.49–0.70) and 2.1 (95% CI: 1.6–2.9) [18,20,22,23,24,25].

The highest pooled negative likelihood ratio corresponded to the Sheppard protocol 0.45 (95% CI: 0.24–0.85) [18,21,23,25], and the highest pooled diagnostic odds ratio was attributed to the Mangham protocol 9 (95% CI: 2–55) [18,21,23,24,25]. 

**Figure 2:**
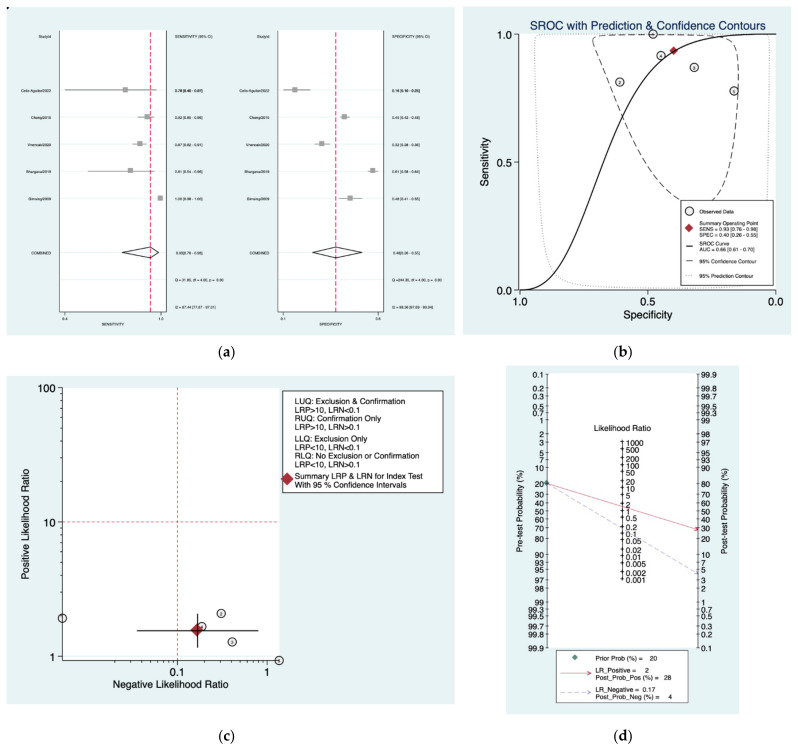
Meta-analysis of Mangham protocol. (**a**) Forrest plot of pooled sensitivity and specificity [8,21,22,23,24,25]; (**b**) Hierarchical summary receiver operating characteristic (HSROC) curve; (**c**) Likelihood ratio scattergram; (**d**) Fagan nomogram.

Figure 2a–d is a graphical representation of the diagnostic accuracy of the Mangham protocol. We have identified a great heterogeneity among studies regarding the reporting of sensitivity (I^2^: 87.4%) and specificity (I^2^: 98.3%) of this protocol (Figure 2a). The area under the curve (AUC) for this protocol was 0.66 (95%CI: 0.61–0.70) (Figure 2b). The likelihood ratio scattergram (Figure 2c) indicated that this protocol is comprised in the right lower quadrant and that it could not be used for exclusion or confirmation of the disease. Finally, the Fagan nomogram (Figure 2d) revealed that, for a given pre-test probability of 20% of vestibular schwannoma, the post-test probability for positive and negative diagnosis of this protocol was 28 and 4%, respectively.

**Figure 3:**
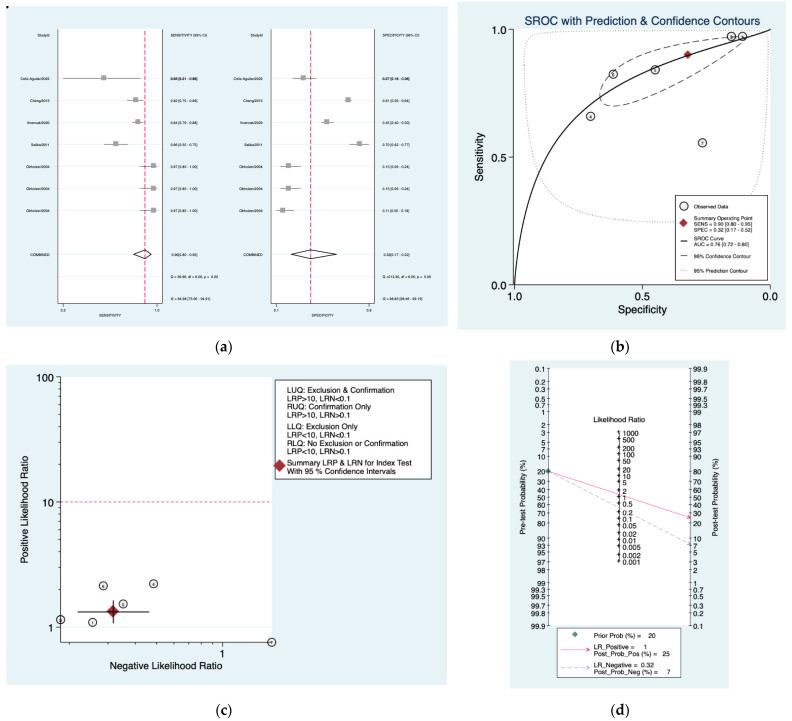
Meta-analysis of Sunderland protocol. (**a**) Forrest plot of pooled sensitivity and specificity [8,10,22,24,25]; (**b**) Hierarchical summary receiver operating characteristic (HSROC) curve; (**c**) Likelihood ratio scattergram; (**d**) Fagan nomogram.

Figure 3a–d is a graphical representation of the diagnostic accuracy of the Sunderland protocol. We have identified a great heterogeneity among studies regarding the reporting of sensitivity (I^2^: 84.9%) and specificity (I^2^: 98.3%) of this protocol (Figure 3a). The area under the curve (AUC) for this protocol was 0.76 (95%CI: 0.72–0.80) (Figure 3b). The likelihood ratio scattergram (Figure 3c) indicated that this protocol is comprised in the right lower quadrant and that it could not be used for exclusion or confirmation of the disease. Finally, the Fagan nomogram (Figure 3d) revealed that, for a given pre-test probability of 20% of vestibular schwannoma, the post-test probability for positive and negative diagnosis of this protocol was 25 and 7%, respectively.

**Figure 4:**
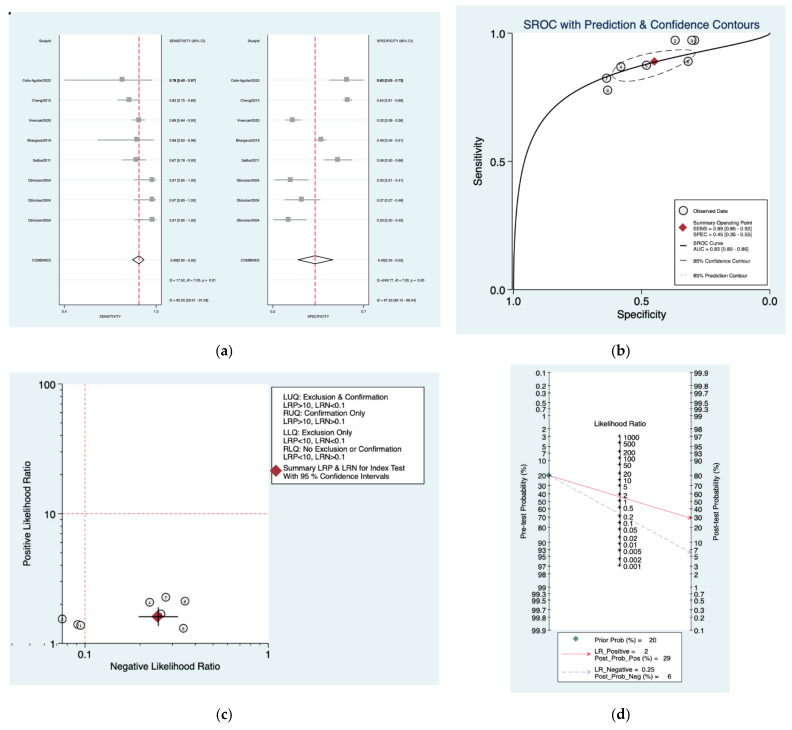
Meta-analysis of Department of Health protocol. (**a**) Forrest plot of pooled sensitivity and specificity [8,10,22,23,24,25]; (**b**) Hierarchical summary receiver operating characteristic (HSROC) curve; (**c**) Likelihood ratio scattergram; (**d**) Fagan nomogram.

Figure 4a–d is a graphical representation of the diagnostic accuracy of the Department of Health protocol. We have identified a great heterogeneity among studies regarding the reporting of sensitivity (I^2^: 60%) and specificity (I^2^: 97.2%) of this protocol (Figure 4a). The area under the curve (AUC) for this protocol was 0.83 (95%CI: 0.80–0.86) (Figure 4b). The likelihood ratio scattergram (Figure 4c) indicated that this protocol is comprised in the right lower quadrant and that it could not be used for exclusion or confirmation of the disease. Finally, the Fagan nomogram (Figure 4d) revealed that, for a given pre-test probability of 20% of vestibular schwannoma, the post-test probability for positive and negative diagnosis of this protocol was 29 and 6%, respectively.

**Figure 5:**
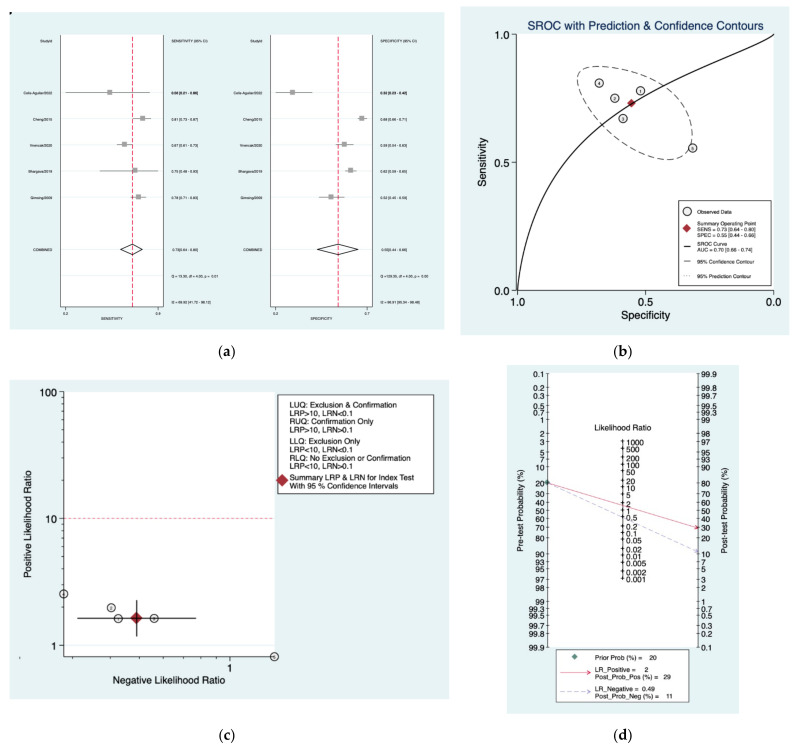
Meta-analysis of Schlauch and Levine protocol. (**a**) Forrest plot of pooled sensitivity and specificity [8,21,23,24,25]; (**b**) Hierarchical summary receiver operating characteristic (HSROC) curve; (**c**) Likelihood ratio scattergram; (**d**) Fagan nomogram.

Figure 5a–d is a graphical representation of the diagnostic accuracy of the Schlauch and Levine protocol. We have identified a great heterogeneity among studies regarding the reporting of sensitivity (I^2^: 69.9%) and specificity (I^2^: 96.9%) of this protocol (Figure 5a). The area under the curve (AUC) for this protocol was 0.70 (95%CI: 0.66–0.74) (Figure 5b). The likelihood ratio scattergram (Figure 5c) indicated that this protocol is comprised in the right lower quadrant and that it could not be used for exclusion or confirmation of the disease. Finally, the Fagan nomogram (Figure 5d) revealed that, for a given pre-test probability of 20% of vestibular schwannoma, the post-test probability for positive and negative diagnosis of this protocol was 29 and 11%, respectively.

**Figure 6:**
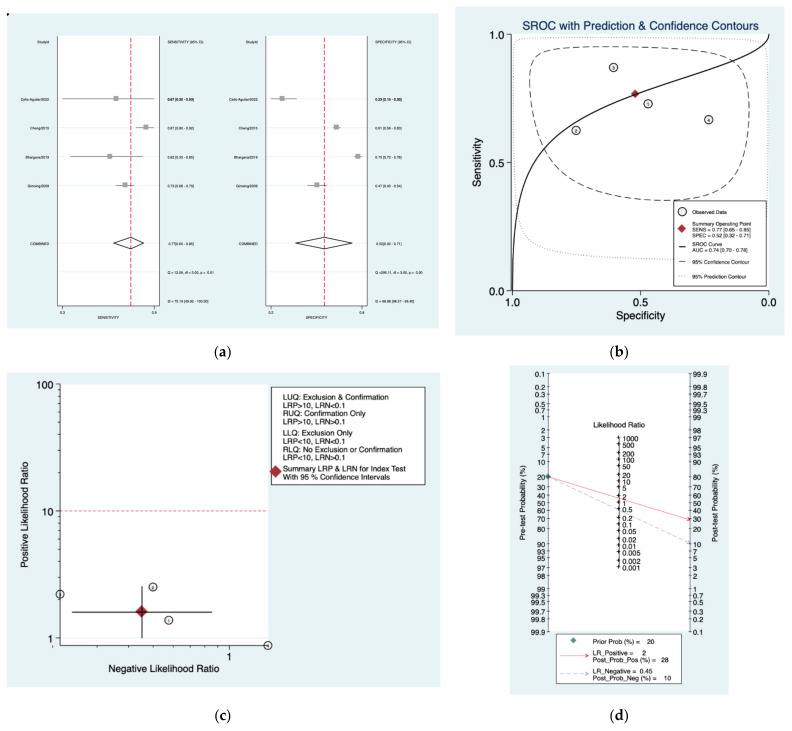
Meta-analysis of Sheppard protocol. (**a**) Forrest plot of pooled sensitivity and specificity [8,21,23,25]; (**b**) Hierarchical summary receiver operating characteristic (HSROC) curve; (**c**) Likelihood ratio scattergram; (**d**) Fagan nomogram.

Figure 6a–d is a graphical representation of the diagnostic accuracy of the Sheppard protocol. We have identified a great heterogeneity among studies regarding the reporting of sensitivity (I^2^: 75.1%) and specificity (I^2^: 98.9%) of this protocol (Figure 6a). The area under the curve (AUC) for this protocol was 0.74 (95%CI: 0.70–0.78) (Figure 6b). The likelihood ratio scattergram (Figure 6c) indicated that this protocol is comprised in the right lower quadrant and that it could not be used for exclusion or confirmation of the disease. Finally, the Fagan nomogram (Figure 6d) revealed that, for a given pre-test probability of 20% of vestibular schwannoma, the post-test probability for positive and negative diagnosis of this protocol was 28 and 10%, respectively.

**Figure 7:**
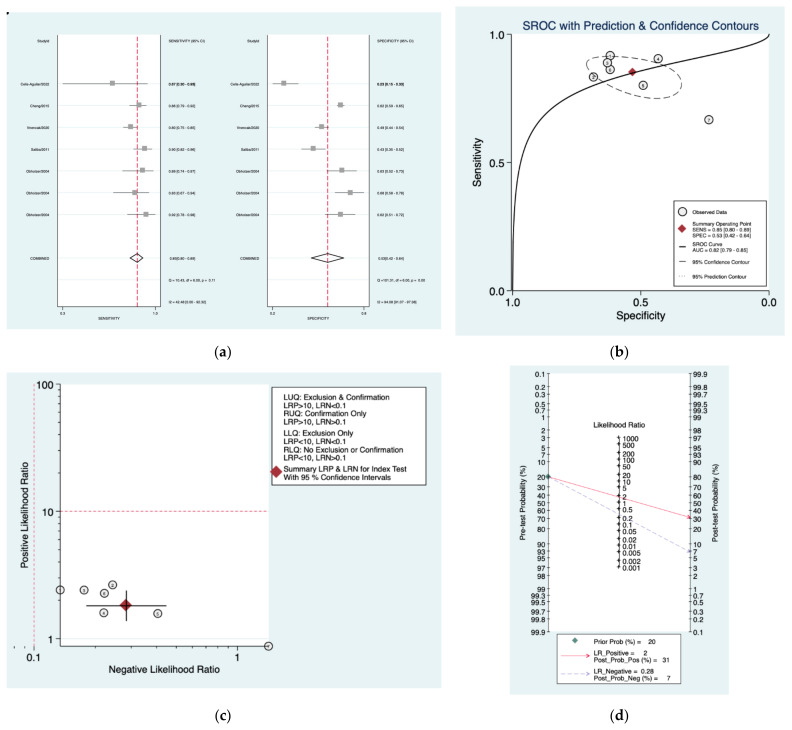
Meta-analysis of Seattle protocol. (**a**) Forrest plot of pooled sensitivity and specificity [8,10,22,24,25]; (**b**) Hierarchical summary receiver operating characteristic (HSROC) curve; (**c**) Likelihood ratio scattergram; (**d**) Fagan nomogram.

Figure 7a–d is a graphical representation of the diagnostic accuracy of the Seattle protocol. We have identified a great heterogeneity among studies regarding the reporting of sensitivity (I^2^: 42.4%) and specificity (I^2^: 94%) of this protocol (Figure 7a). The area under the curve (AUC) for this protocol was 0.82 (95%CI: 0.79–0.85) (Figure 7b). The likelihood ratio scattergram (Figure 7c) indicated that this protocol is comprised in the right lower quadrant and that it could not be used for exclusion or confirmation of the disease. Finally, the Fagan nomogram (Figure 7d) revealed that, for a given pre-test probability of 20% of vestibular schwannoma, the post-test probability for positive and negative diagnosis of this protocol was 31 and 7%, respectively.

**Figure 8:**
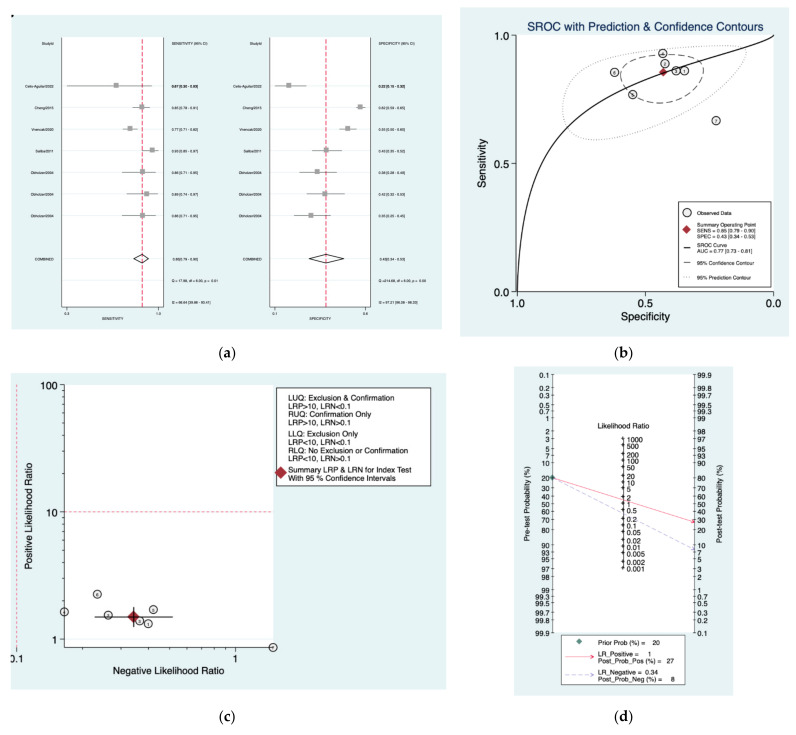
Meta-analysis of Oxford protocol. (**a**) Forrest plot of pooled sensitivity and specificity [8,10,22,24,25]; (**b**) Hierarchical summary receiver operating characteristic (HSROC) curve; (**c**) Likelihood ratio scattergram; (**d**) Fagan nomogram.

Figure 8a–d is a graphical representation of the diagnostic accuracy of the Oxford protocol. We have identified a great heterogeneity among studies regarding the reporting of sensitivity (I^2^: 66.6%) and specificity (I^2^: 97.2%) of this protocol (Figure 8a). The area under the curve (AUC) for this protocol was 0.77 (95%CI: 0.73–0.81) (Figure 8b). The likelihood ratio scattergram (Figure 8c) indicated that this protocol is comprised in the right lower quadrant and that it could not be used for exclusion or confirmation of the disease. Finally, the Fagan nomogram (Figure 8d) revealed that, for a given pre-test probability of 20% of vestibular schwannoma, the post-test probability for positive and negative diagnosis of this protocol was 27 and 8%, respectively.

**Figure 9:**
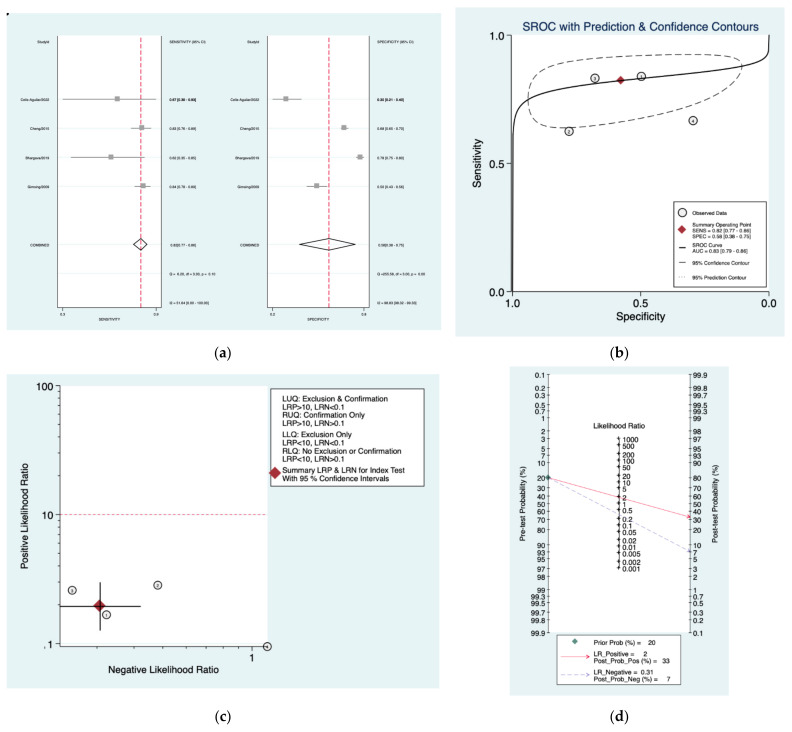
Meta-analysis of Obholzer protocol. (**a**) Forrest plot of pooled sensitivity and specificity [8,21,23,25]; (**b**) Hierarchical summary receiver operating characteristic (HSROC) curve; (**c**) Likelihood ratio scattergram; (**d**) Fagan nomogram.

Figure 9a–d is a graphical representation of the diagnostic accuracy of the Obholzer protocol. We have identified a great heterogeneity among studies regarding the reporting of sensitivity (I^2^: 51.6%) and specificity (I^2^: 98.8%) of this protocol (Figure 9a). The area under the curve (AUC) for this protocol was 0.83 (95%CI: 0.79–0.86) (Figure 9b). The likelihood ratio scattergram (Figure 9c) indicated that this protocol is comprised in the right lower quadrant and that it could not be used for exclusion or confirmation of the disease. Finally, the Fagan nomogram (Figure 9d) revealed that, for a given pre-test probability of 20% of vestibular schwannoma, the post-test probability for positive and negative diagnosis of this protocol was 33 and 7%, respectively.

**Figure 10:**
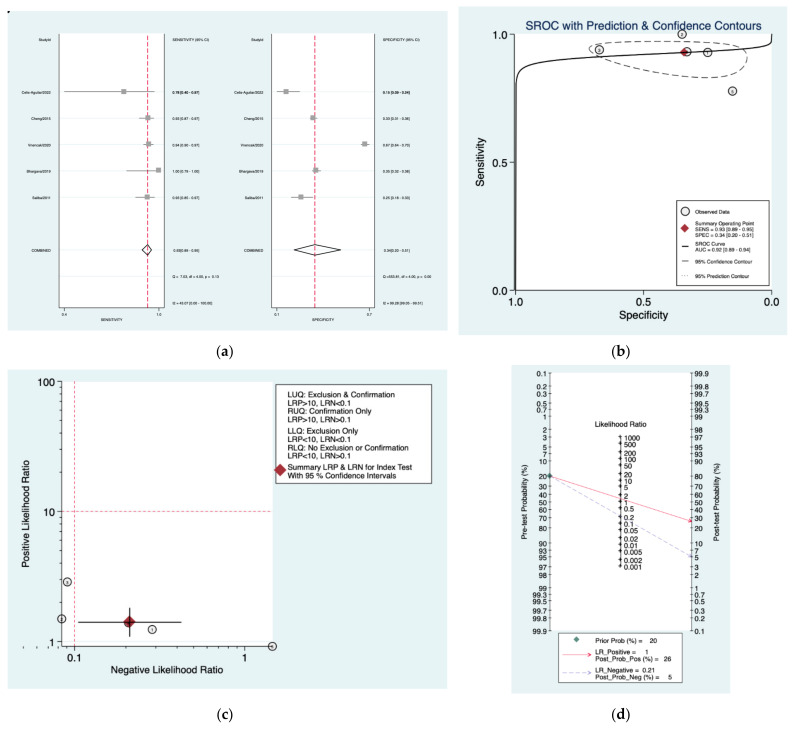
Meta-analysis of Amclass protocol. (**a**) Forrest plot of pooled sensitivity and specificity [8,22,23,24,25]; (**b**) Hierarchical summary receiver operating characteristic (HSROC) curve; (**c**) Likelihood ratio scattergram; (**d**) Fagan nomogram.

Figure 10a–d is a graphical representation of the diagnostic accuracy of the Amclass protocol. We have identified a great heterogeneity among studies regarding the reporting of sensitivity (I^2^: 43%) and specificity (I^2^: 99.2%) of this protocol (Figure 10a). The area under the curve (AUC) for this protocol was 0.92 (95%CI: 0.89–0.94) (Figure 10b). The likelihood ratio scattergram (Figure 10c) indicated that this protocol is comprised in the right lower quadrant and that it could not be used for exclusion or confirmation of the disease. Finally, the Fagan nomogram (Figure 10d) revealed that, for a given pre-test probability of 20% of vestibular schwannoma, the post-test probability for positive and negative diagnosis of this protocol was 26 and 5%, respectively.

**Figure 11:**
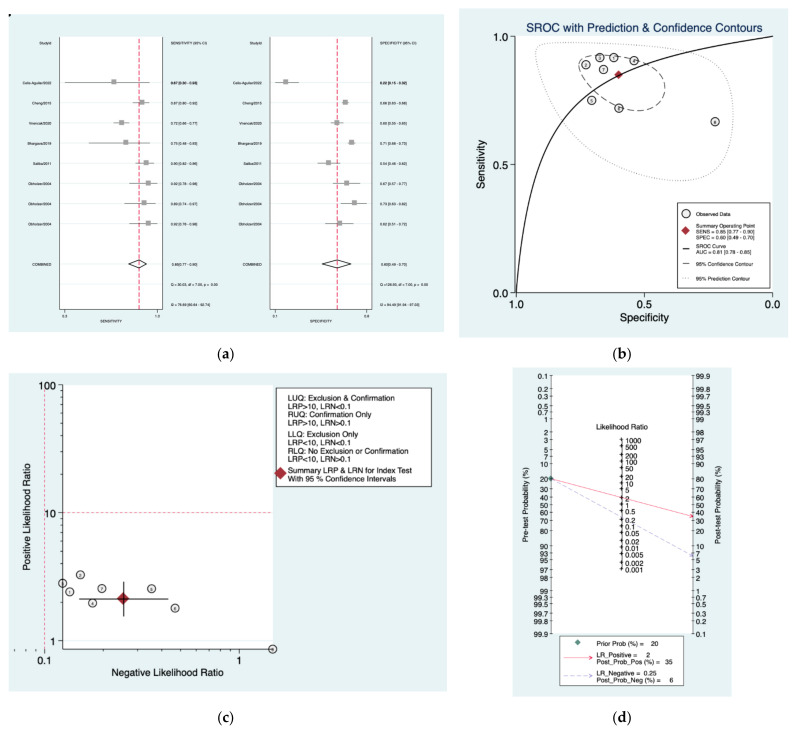
Meta-analysis of AAO protocol. (**a**) Forrest plot of pooled sensitivity and specificity [8,10,22,23,24,25]; (**b**) Hierarchical summary receiver operating characteristic (HSROC) curve; (**c**) Likelihood ratio scattergram; (**d**) Fagan nomogram.

Figure 11a–d is a graphical representation of the diagnostic accuracy of the AAO protocol. We have identified a great heterogeneity among studies regarding the reporting of sensitivity (I^2^: 76.6%) and specificity (I^2^: 94.4%) of this protocol (Figure 11a). The area under the curve (AUC) for this protocol was 0.81 (95%CI: 0.78–0.86) (Figure 11b). The likelihood ratio scattergram (Figure 11c) indicated that this protocol is comprised in the right lower quadrant and that it could not be used for exclusion or confirmation of the disease. Finally, the Fagan nomogram (Figure 11d) revealed that, for a given pre-test probability of 20% of vestibular schwannoma, the post-test probability for positive and negative diagnosis of this protocol was 35 and 6%, respectively.

**Figure 12:**
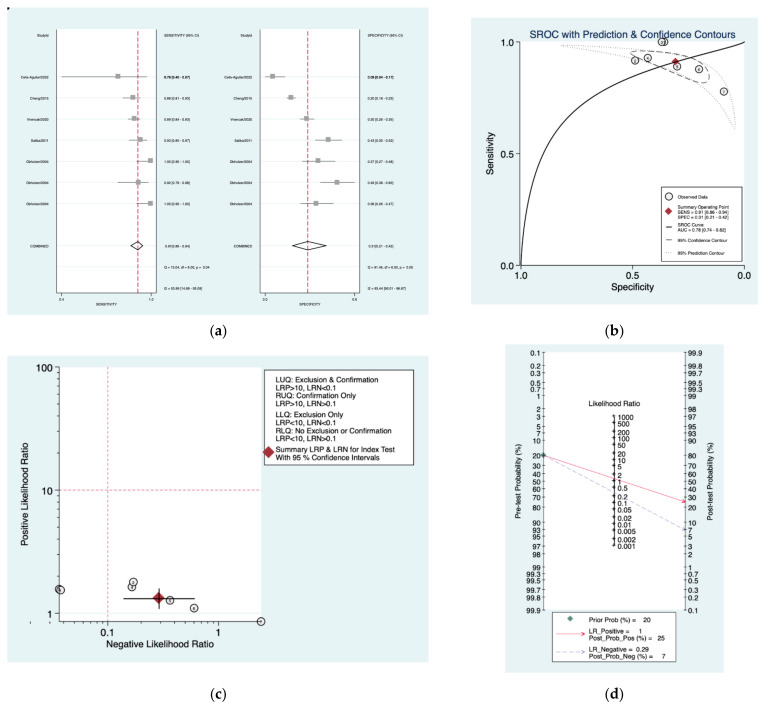
Meta-analysis of Nashville protocol. (**a**) Forrest plot of pooled sensitivity and specificity [8,10,22,24,25]; (**b**) Hierarchical summary receiver operating characteristic (HSROC) curve; (**c**) Likelihood ratio scattergram; (**d**) Fagan nomogram.

Figure 12a–d is a graphical representation of the diagnostic accuracy of the Nashville protocol. We have identified a great heterogeneity among studies regarding the reporting of sensitivity (I^2^: 53.9%) and specificity (I^2^: 93.4%) of this protocol (Figure 11a). The area under the curve (AUC) for this protocol was 0.78 (95%CI: 0.74–0.82) (Figure 11b). The likelihood ratio scattergram (Figure 11c) indicated that this protocol is comprised in the right lower quadrant and that it could not be used for exclusion or confirmation of the disease. Finally, the Fagan nomogram (Figure 11d) revealed that, for a given pre-test probability of 20% of vestibular schwannoma, the post-test probability for positive and negative diagnosis of this protocol was 35 and 6%, respectively.

## 4. Discussion

This systematic review and meta-analysis evaluated the diagnostic accuracy of 11 pure-tone audiometry protocols, which were previously reported to the gold standard examination—MRI, for the diagnosis of vestibular schwannoma in patients with unilateral hearing loss. As the incidence rate of this condition is following an ascending trend [9], patient selection and their risk stratification becomes more important to clinicians. 

Our results showed that the pooled sensitivity of these protocols was good, ranging between 0.73 and 0.93, with the highest values achieved by Mangham (0.93), Amclass (0.93), and Nashville (0.91) protocols. On the other hand, the specificity of the evaluated protocols was heterogeneous and low, ranging from 0.31 (Nashville) to 0.60 (AAO). 

Our study results revealed good values for the HSROC curve, ranging from 0.66 (Mangham) to 0.92 (Amclass). Nonetheless, all protocols were located in the right lower quadrant on the likelihood scattergram, which indicated that none of them could be used for exclusion or confirmation of the disease. Moreover, the post-test probabilities for positive and negative diagnosis of these protocols were extremely low. 

These arguments support the hypothesis that the evaluated pure-tone audiometry protocols cannot be used for a proper screening or diagnosis of vestibular schwannoma despite of their good sensitivity. Thus, MRI investigation remains the gold standard for the evaluation of patients with unilateral hearing loss, even though its costs are high [35,36,37]. 

Even though PTA protocols could be used in low-resource medical settings due to their high sensibility, simplicity, objectivity, easiness to apply, and low costs, clinicians must take into consideration their low specificity, which may give a high number of false positives when evaluating patients with unilateral hearing loss [38]. Moreover, the European Association of Neuro-Oncology (EANO) recommends annual follow-up with microbeam radiation therapy and audiometry in patients with conservatively treated, radiated, and incompletely resected VS [39]. 

In recent years, data collected from PTA along with the patient’s clinical characteristics were incorporated into algorithms for the prediction of the need for active treatment with approximately 90% accuracy [40]. This paves the way for a perspective surrounding the improvement of PTA protocols that would result in a higher specificity of the tests, and thus to a better patient selection. 

Our results are comparable to those reported in a 2017 systematic review and meta-analysis that evaluated the diagnostic accuracy of different non-imaging screening protocols that can be used to select patients at high risk of VS [41]. The authors indicated good sensitivity (88–91%) but low specificity (31–58%) for the analyzed protocols. Despite the heterogeneity of the reported data, its results constitute another argument that favors the use of MRI for the evaluation of patients with unilateral hearing loss.

The results from this meta-analysis should be evaluated considering some inherent limitations. First of all, we could not assess all the published PTA protocols because the limited information extracted from the included studies did not allow a coherent statistical analysis. Secondly, we could not include randomized controlled trials in this study, and the results are based on observational studies, such as cohort, cross-sectional, or case-control. Thirdly, we did find a high degree of heterogeneity regarding the reporting of sensitivity and specificity data. All these limitations could derive from the disparity of data reported in the literature about the topic. Moreover, it is expected that PTA protocols will be updated based on the data emerging from the new integrative technologies that use artificial intelligence [42,43]. 

Further studies, on larger cohorts of patients, or several randomized controlled trials could represent scientific material of higher quality for the next meta-analysis. Meanwhile, we consider that our results support the use of pure-tone audiometry protocols in low resource settings, at least for the risk stratification of patients with asymmetric hearing loss and a high degree of suspicion for vestibular schwannoma. Newer technologies, such as those based on artificial intelligence and machine learning techniques, could help in the process of risk stratification of patients who have a high risk of developing vestibular schwannoma [44,45,46,47]. 

## Figures and Tables

**Table 1:** Characteristics of the included studies.

Author	Year	Study Design	Number of Patients Included	Index Tests
Obholzer et al. [20]	2004	Case-control	128	9 PTA protocols
Gimsing [21]	2009	Case-control	424	PTA shapes 8 PTA protocols
Saliba et al. [22]	2011	Cohort	212	8 PTA protocols
Cheng et al. [18]	2015	Cohort	1751	15 PTA protocols
Bhargava et al. [23]	2019	Cross-sectional	1059	14 PTA protocols
Vnencak et al. [24]	2020	Case-control	688	14 PTA protocols
Celis-Aguilar et al. [25]	2022	Cross-sectional	107	15 PTA protocols

Legend: PTA—pure tone audiometry.

**Table 2:** Quality assessment of the included studies.

Study	Risk of Bias				Applicability Concerns		
	Patient Selection	Index Test	Reference Standard	Flow and Timing	Patient Selection	Index Test	Reference Standard
Gimsing	?	?	☺	?	☺	☺	?
Obholzer et al. [20]	?	?	?	?	☺	☺	☺
Saliba et al. [22]	☺	☺	☺	?	☺	☺	☺
Bhargava et al. [23]	?	?	?	?	?	☺	☺
Vnencak et al. [24]	☹	?	?	?	?	☺	☺
Cheng et al. [18]	?	?	?	?	☺	?	☺
Celis-Aguilar et al. [25]	☹	☺	?	?	?	☺	☺

Legend: ☺Low Risk; ☹High Risk; ? Unclear Risk.

**Table 3:** Pooled estimates of diagnostic accuracy parameters for the included PTA protocols.

PTA Protocol	Sensitivity (Pooled Estimate/95% CI)	Specificity (Pooled Estimate/95% CI)	Positive Likelihood Ratio (Pooled Estimate/95% CI)	Negative Likelihood Ratio (Pooled Estimate/95% CI)	Diagnostic Odds Ratio (Pooled Estimate/95% CI)
Mangham [26]	0.93 (0.76–0.98)	0.40 (0.26–0.55)	1.5 (1.2–2.1)	0.17 (0.04–0.79)	9 (2–55)
Sunderland [27]	0.90 (0.80–0.95)	0.32 (0.17–0.52)	1.3 (1.1–1.6)	0.32 (0.22–0.46)	4 (3–7)
DOH [28]	0.89 (0.85–0.92)	0.45 (0.35–0.55)	1.6 (1.4–1.9)	0.25 (0.20–0.32)	6 (5–9)
Schlauch and Levine [29]	0.73 (0.64–0.80)	0.55 (0.44–0.66)	1.6 (1.2–2.3)	0.49 (0.31–0.77)	3 (2–7)
Sheppard [30]	0.77 (0.65–0.85)	0.52 (0.32–0.71)	1.6 (1.0–2.5)	0.45 (0.24–0.85)	4 (3–10)
Seattle [31]	0.85 (0.80–0.89)	0.53 (0.42–0.64)	1.8 (1.4–2.4)	0.28 (0.18–0.45)	6 (3–13)
Oxford [30]	0.85 (0.79–0.90)	0.43 (0.34–0.53)	1.5 (1.3–1.8)	0.34 (0.23–0.52)	4 (3–8)
Obholzer [20]	0.82 (0.77–0.86)	0.58 (0.38–0.75)	1.9 (1.3–3.0)	0.31 (0.23–0.42)	6 (3–13)
Amclass [32]	0.93 (0.89–0.95)	0.34 (0.20–0.51)	1.4 (1.1–1.8)	0.21 (0.11–0.42)	7 (3–17)
AAO [33]	0.85 (0.77–0.90)	0.60 (0.49–0.70)	2.1 (1.6–2.9)	0.25 (0.15–0.43)	8 (4–18)
Nashville [34]	0.91 (0.86–0.94)	0.31 (0.21–0.42)	1.3 (1.1–1.6)	0.29 (0.14–0.61)	5 (4–11)

Legend: CI—confidence interval; PTA—pure tone audiometry; AAO—American Academy of Otolaryngology protocol; DOH—Department of Health.

## Data Availability

This systematic review and meta-analysis is registered in the Open Science Framework Registry (DOI: 10.17605/OSF.IO/FRGTC (accessed on 9 October 2022)), and it is available at: https://archive.org/details/osf-registrations-frgtc-v1 (accessed on 9 October 2022). Additional data is available upon reasonable request from the corresponding author due to local policies.

## Supplementary Materials

The following supporting information can be downloaded at: https://www.mdpi.com/article/10.3390/diagnostics12112776/s1, S1: Embase Search Strategy; S2: Medline Search Strategy; S3: Cochrane Search Strategy; Table S1: Definition of asymmetrical hearing loss for the evaluated pure-tone audiometry protocols.
