# Supplementary material for: The Diagnostic Accuracy of Pure-Tone Audiometry Screening Protocols for Vestibular Schwannoma in Patients with Asymmetrical Hearing Loss—A Systematic Review and Meta-Analysis
Source: Diagnostics (Basel). 2022 Nov 14;12(11):2776. doi: 10.3390/diagnostics12112776 (PMC9689241; doi:10.3390/diagnostics12112776)
Supplement: Supplementary file 1 [file diagnostics-12-02776-s001.zip › S3. Cochrane Search Strategy.pdf]

Search Name: Cochrane Search

ID Search Hits

#1 (acustic neuroma):ti,ab,kw OR (acustic neurinoma):ti,ab,kw OR ("vestibular schwannoma"):ti,ab,kw AND (MRI):ti,ab,kw AND ("pure tone audiometry"):ti,ab,kw (Word variations have been searched) 5
