# Supplementary material for: The Diagnostic Accuracy of Pure-Tone Audiometry Screening Protocols for Vestibular Schwannoma in Patients with Asymmetrical Hearing Loss—A Systematic Review and Meta-Analysis
Source: Diagnostics (Basel). 2022 Nov 14;12(11):2776. doi: 10.3390/diagnostics12112776 (PMC9689241; doi:10.3390/diagnostics12112776)
Supplement: Supplementary file 1 [file diagnostics-12-02776-s001.zip › Table S1. Definition of ASHL for different PTA protocols.pdf]

Table S1. Definition of **asymmetrical hearing loss for the evaluated pure-tone audiometry protocols.**

**PTA protocol**	**Definition of asymetrical hearing loss**
Mangham	Average asymmetry >5 dB (1–8 kHz)
Sunderland	≥20 dB at two neighboring frequencies
Department of Health	≥20 dB at any single frequency between 0.5 and 4 kHz
Schlauch and Levine	Males: average Asymmetry >19 dB (1–8 kHz) Females: asymmetry at 4 kHz >19 dB
Sheppard	Average asymmetry >14 dB (0.25–8 kHz) or normal hearing with unilateral tinnitus or canal paresis
Seattle	≥15 dB between the average of 1 and 8 kHz
Oxford	≥15 dB between the average of 0.5 and 8 kHz
Obholzer	If better ear hearing level B <31 dB, asymmetry >15 dB at two neighbouring frequencies (0.25–8 kHz). Otherwise, asymmetry >20 dB
Amclass	≥10 dB at two or more frequencies; OR ≥15 dB at any single frequency
AAO	≥15 dB between the average of 0.5, 1, 2 and 3 kHz
Nashville	≥15 dB at any single frequency between 0.5 and 4 kHz
